# Supplementary figures and images for: Loss of RNase J leads to multi-drug tolerance and accumulation of highly structured mRNA fragments in Mycobacterium tuberculosis
Source: PLoS Pathog. 2022 Jul 13;18(7):e1010705. doi: 10.1371/journal.ppat.1010705 (PMC9312406; doi:10.1371/journal.ppat.1010705)

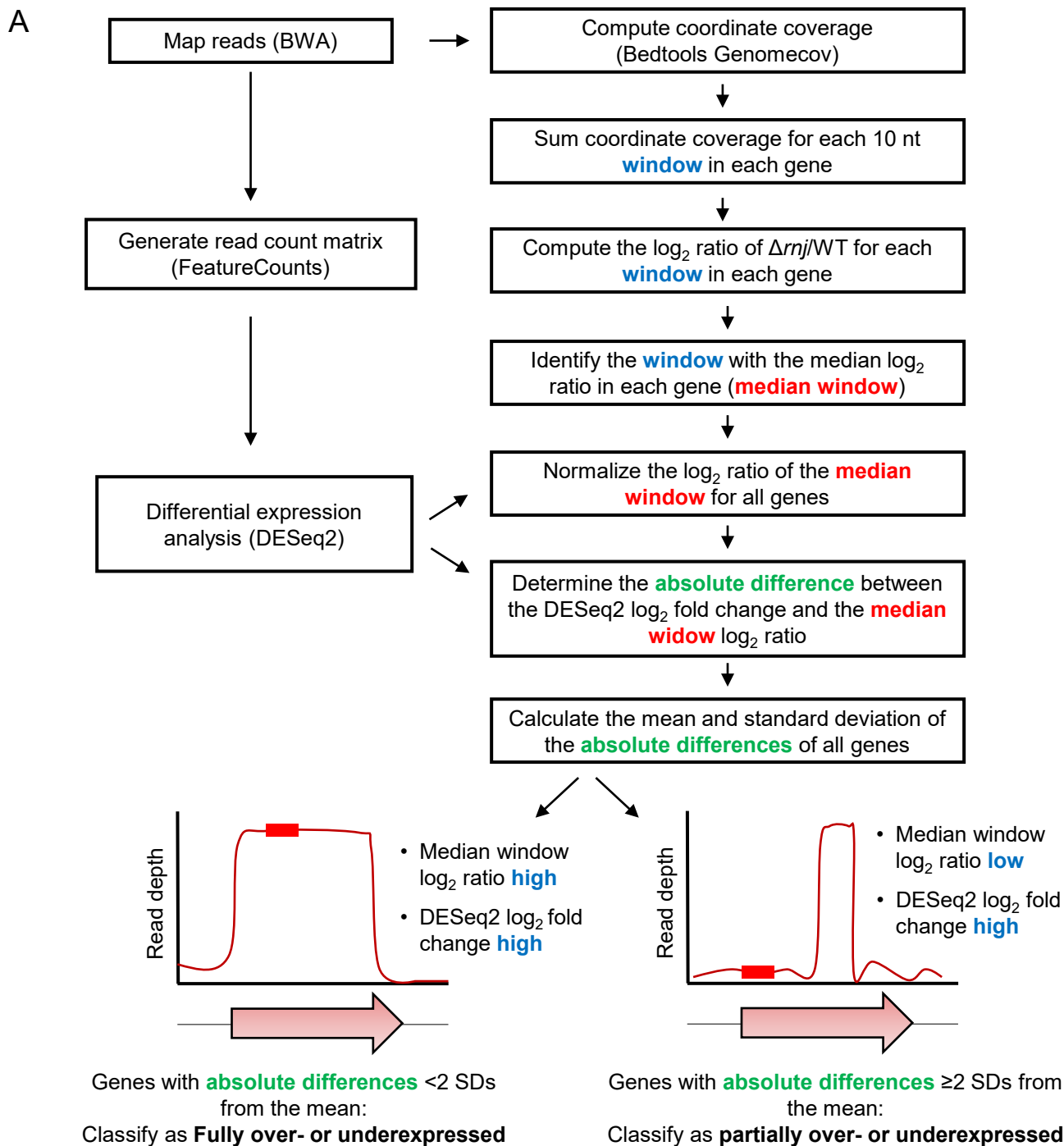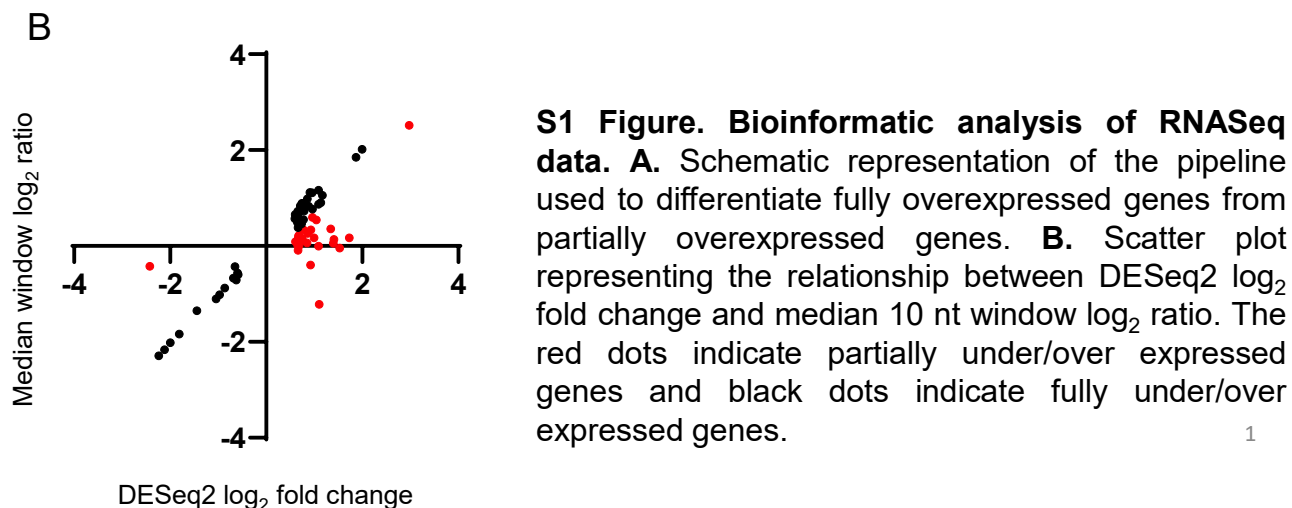

Supplement: S1 Fig — (PDF) [file ppat.1010705.s007.pdf]
