# Supplementary material for: Loss of RNase J leads to multi-drug tolerance and accumulation of highly structured mRNA fragments in Mycobacterium tuberculosis
Source: PLoS Pathog. 2022 Jul 13;18(7):e1010705. doi: 10.1371/journal.ppat.1010705 (PMC9312406; doi:10.1371/journal.ppat.1010705)
Supplement: S2 Fig — (PDF) [file ppat.1010705.s008.pdf]

```
Mtb     ------------MDVDLPPPGPLTSGGLRVTALGGINEIGRNMTVFEHLGRLLIIDCGVL    48
Msm     ------------MSAELAPPPPLAPGGLRVTALGGISEIGRNMTVFEHLGRLLIVDCGVL    48
Sco     ---------MSHPHPELGRPPALPKGGLRVTPLGGLGEIGRNMTVFEYGGRLLIVDCGVL    51
Tth     MENQERKPRRRRRRRPQEGSQGGPQDHVEIIPLGGMGEIGKNITVFRFRDEIFVLDGGLA    60
Dra     ---------MTRPEQPRPESADLPAPTLEVIPLGGMGEIGKNITVFRYGDEIVVVDGGLA    51
                                   :.:  ***:.***:*:***.. ..:.::* *:

Mtb     FPGHDEPGVDLILPDMRHVEDRLDDIEALVLTHGHEDHIGAIPFL----LKLRPDIPVVG    104
Msm     FPGHDEPGVDLILPDLRHIEDRLDEIEALVVTHAHEDHIGAIPFL----LKLRPDIPVVG    104
Sco     FPEEEQPGIDLILPDFTSIRDRLDDIEGIVLTHGHEDHIGGVPFL----LREKPDIPLIG    107
Tth     FPEEGMPGVDLLIPRVDYLIEHRHKIKAWVLTHGHEDHIGGLPFLLPMIFGKESPVPIYG    120
Dra     FPKAHQMGIDLIVPRIDYLLEHQDKIKGWILTHGHEDHIGGLPYI----FARLPRVPVYG    107
        **     *:**::* .  : :: ..*:. ::**.******.:*::    :     :*: *

Mtb     SKFTLALVAEKCREYRITPVFVEVRE---GQSTRHG-VFECEYFAVNHSTPDALAIAVYT    160
Msm     SKFTIALVREKCREHRLKPKFVEVAE---RQSSQHG-VFECEYFAVNHSIPGCLAVAIHT    160
Sco     SKLTLALIEAKLQEHRIRPYTLEVAE---GHRERVG-PFDCEFVAVNHSIPDALAVAIRT    163
Tth     ARLTLGLLRGKLEEFGLRPGAFNLKEISPDDRIQVGRYFTLDLFRMTHSIPDNSGVVIRT    180
Dra     LPLTLALVREKLSEFGLQD--VDLREVTYGDEVRFGQSFVAEFFCMTHSIPDNAGYILKT    165
          :*:.*:  *  *. :    .:: *    .  : *  *  : . :.** *.  .  : *

Mtb     GAGTILHTGDIKFDQLPP--DGRPTDLPGMSRLGDTGVDLLLCDSTNAEIPGVGPSESEV    218
Msm     GAGTVLHTGDIKLDQLPL--DGRPTDLPGMSRLGDAGVDLFLCDSTNSEHPGVSPSESEV    218
Sco     PAGMVVHTGDFKMDQLPL--DGRLTDLHAFARLSEEGIDLLLADSTNAEVPGFVPPERDI    221
Tth     PIGTIVHTGDFKLDPTPI--DGKVSHLAKVAQAGAEGVLLLIADATNAERPGYTPSEMEI    238
Dra     PVGDVLHTGDFKIDPDVGTGAGIVSDLERVEQAGKDGVLLLISDSTNAERPGHTPSEAEI    225
          * ::****:*:*       *  :.*  . : .  *: *::.*:**:* **  * * ::

Mtb     GPTLHRLIRGADGRVIVACFASNVDRVQQIIDAAVALGRRVSFVGRSMVRNMRVARQLGF    278
Msm     GPTLHRLIRGAEGRVIVACFASNVDRVQQIIDAAVALGRRVSFVGRSMVRNMGIARELGY    278
Sco     SNVLRQVFANARKRIIVASFASHVHRIQQILDAAHEYGRRVAFVGRSMVRNMGIARDLGY    281
Tth     AKELDRVIGRAPGRVFVTTFASHIHRIQSVIWAAEKYGRKVAMEGRSMLKFSRIALELGY    298
Dra     ARNLEEIIKGCRGRVFLTTFASQVYRIQNILDLAHRQGRRVVMEGRSMIKYAQAAQATGH    285
        .  * .::  .  *:::: ***:: *:*.::  *   **:* : ****::    *   *.

Mtb     LRVADSDLIDIAAAETMAPDQVVLITTGTQGEPMSALSRMSRGEHRSITLTAGDLIVLSS    338
Msm     LKVDDSDILDIAAAEMMPPDRVVLITTGTQGEPMAALSRMSRGEHRSITLTSGDLIILSS    338
Sco     LKVPPGLVVDVKTLDDLPDSEVVLVCTGSQGEPMAALSRMANRDHQ-IRIVNGDTVILAS    340
Tth     LKVKDRL-YTLEEVKDLPDHQVLILATGSQGQPMSVLHRLAFEGHAKMAIKPGDTVILSS    357
Dra     MNPPEPF-LTSEEVGELQDQQVLFVCTGSQGQPMAVLGRLAFGTHAKIALRRGDTVILSS    344
        :.              :   .*::: **:**:**:.* *::   *  : :  ** ::*:*

Mtb     SLIPGNEEAVFGVIDALSKIGARVVTNAQARVHVSGHAYAGELLFLYNGVRPRNVMPVHG    398
Msm     SLIPGNEEAVYGVIDSLSKIGARVVTNAQARVHVSGHAYAGELLFLYNGVRPRNVMPVHG    398
Sco     SLIPGNENAVYRVINGLTRWGANVVHKGNAKVHVSGHASAGELLYFYNICRPKNLMPVHG    400
Tth     SPIPGNEEAVNRVINRLYALGAYVLYPPTYKVHASGHASQEELKLILNLTTPRFFLPWHG    417
Dra     NPIPGNEDAVNLIVNRLYEIGVDVVYPPTYRVHASGHASQEELATILNLTRPKFFLPWHG    404
        . *****:**  ::: *   *. *:     :**.****   **  : *   *: .:* **
```

S2 Figure – continued on following page.

```
Mtb      TWRMLRANAKLAASTGVPQESILLAENGVSVDLVAGKASISGAVPVGKMFVDGLIAGDVG      458
Msm      TWRHLRANAALAASTGVPPENIVLAENGVSVDLVAGRASISGAVTVGKMFVDGLITGDVG      458
Sco      EWRHLRANAELGALTGVPHDRIVIAEDGVVVDLVEGKAKITGKVQAGYVYVDGLSVGDVG      460
Tth      EVRHQMNFKWLAESMSRPPEKTLIGENGAVYRLTRETFEKVGEVPHGVLYVDGLGVGDIT      477
Dra      EPRHQINHAKLAQTLPRPPKRTLIAKNGDIVNLGPDEFRVSGTVAAGAVYVDGLGVGDVN      464
           *       *.     * .  ::.::*    *        * *  * ::**** .**:

Mtb      DITLGERLILS-SGFVAVTVVVRRGTGQPLAAPHLHSRGFSEDPKALEPAVRKVEAELES      517
Msm      DATLGERLILS-SGFVSITVVVHRGTGRPAGPAHLISRGFSEDPKALEPVAQKVERELEA      517
Sco      EPALKDRKILGDEGIISVFVVMDSSTGKITGGPHVQARGSGIEDSAFAAVLPKVTEALER      520
Tth      EEILADRRHMAEEGLVVITALAGED-----PVVEVVSRGFVKAGERLLGEVRRMALEALK      532
Dra      DDVLLDRVNLSQEGLLILTAVLHPT-----PHVEVVARGFARPNRDLELQIRRVALEAVE      519
         :  * :*  :. .*:: : .:            .: :**       :     ::

Mtb      LVAANVTDPIRIAQGVRRTVGKWVGETYRRQPMIVPTVIEV        558
Msm      LAADNVTDPTRIAQAVRRTVGKWVGETYRRQPMIVPTVIEI        558
Sco      SAQDGVVEPHQMQQLIRRTLGKWVSDTYRRRPMILPVVVEV        561
Tth      NGVREKKPLERIRDDIYYPVKKFLKKATGRDPMILPVVIEG        573
Dra      QGLREKKRLEDVRDDMYGAVRRFTRKATGRNPVLIPMIVD-        559
                    : : :   : ::  .:  * *:::* :::
```

S2 Figure (continued from previous page). Multiple sequence alignment of RNase J from *M. tuberculosis* (Mtb), *M. smegmatis* (Msm), *Streptomyces coelicolor* (Sco), *Thermus thermophilus* (Tth), and *Deinococcus radiodurans* (Dra). Residues found to be mutated in INH-resistant Mtb clinical strains are highlighted in red. Sequences were aligned by Clustal Omega 1.2.4 (Madeira et al., 2022 [50]).
