# Supplementary material for: Loss of RNase J leads to multi-drug tolerance and accumulation of highly structured mRNA fragments in Mycobacterium tuberculosis
Source: PLoS Pathog. 2022 Jul 13;18(7):e1010705. doi: 10.1371/journal.ppat.1010705 (PMC9312406; doi:10.1371/journal.ppat.1010705)
Supplement: S3 Fig — (PDF) [file ppat.1010705.s009.pdf]

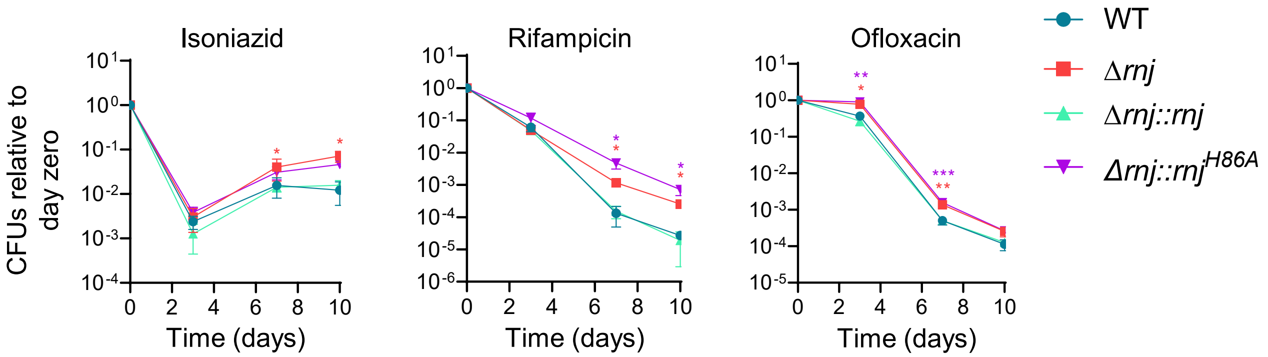

S3 Figure. Loss of RNase J affects drug sensitivity in Mtb H37Rv. Time-kill curves comparing Δ*rnj* transformed with empty vector (pJEB402), Δ*rnj* complemented with the catalytic site mutant *rnj*^{H86A} under the strong UV15 promoter, Δ*rnj*::*rnj*, and the WT strain transformed with empty vector (pJEB402) in the H37Rv background are shown. *$p<0.05$, **$p<0.01$, ***$p<0.001$ two-way ANOVA comparing Δ*rnj* (red stars), Δ*rnj*::*rnj* (green stars) and Δ*rnj*::*rnj*^{H86A} (violet stars) to the WT (control group), with Benjamini and Hochberg FDR 0.05.
