# Supplementary material for: Loss of RNase J leads to multi-drug tolerance and accumulation of highly structured mRNA fragments in Mycobacterium tuberculosis
Source: PLoS Pathog. 2022 Jul 13;18(7):e1010705. doi: 10.1371/journal.ppat.1010705 (PMC9312406; doi:10.1371/journal.ppat.1010705)
Supplement: S4 Fig — (PDF) [file ppat.1010705.s010.pdf]

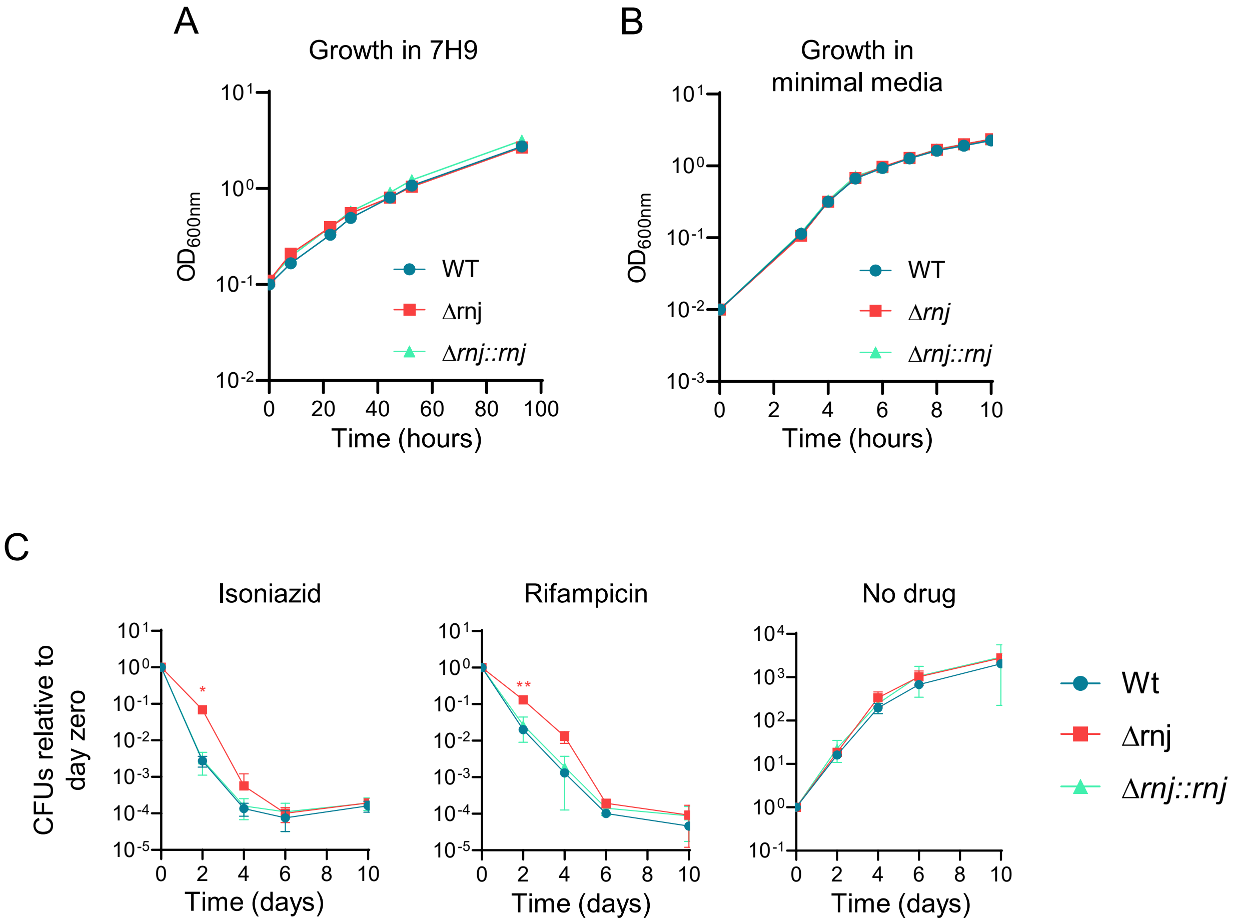

S4 Figure. Growth kinetics in drug-free 7H9 and minimal media and time-kill curves in minimal media. A. Mtb mc[2]6230 WT, *Δrnj,* and Δ*rnj*::*rnj* were grown in 7H9 media, starting with an initial OD_{600nm}=0.1. **B.** Strains were grown in minimal media (initial OD_{600nm}=0.01) supplemented with glycerol and tween-80. **C.** Time-kill curves in minimal media comparing Δ*rnj* and Δ*rnj*::*rnj* to the WT strain in the Mtb mc[2]6230 background are shown. *$p<0.05$, **$p<0.01$ two-way ANOVA with Benjamini and Hochberg FDR 0.05.
