# Supplementary material for: Loss of RNase J leads to multi-drug tolerance and accumulation of highly structured mRNA fragments in Mycobacterium tuberculosis
Source: PLoS Pathog. 2022 Jul 13;18(7):e1010705. doi: 10.1371/journal.ppat.1010705 (PMC9312406; doi:10.1371/journal.ppat.1010705)
Supplement: S5 Fig — (PDF) [file ppat.1010705.s011.pdf]

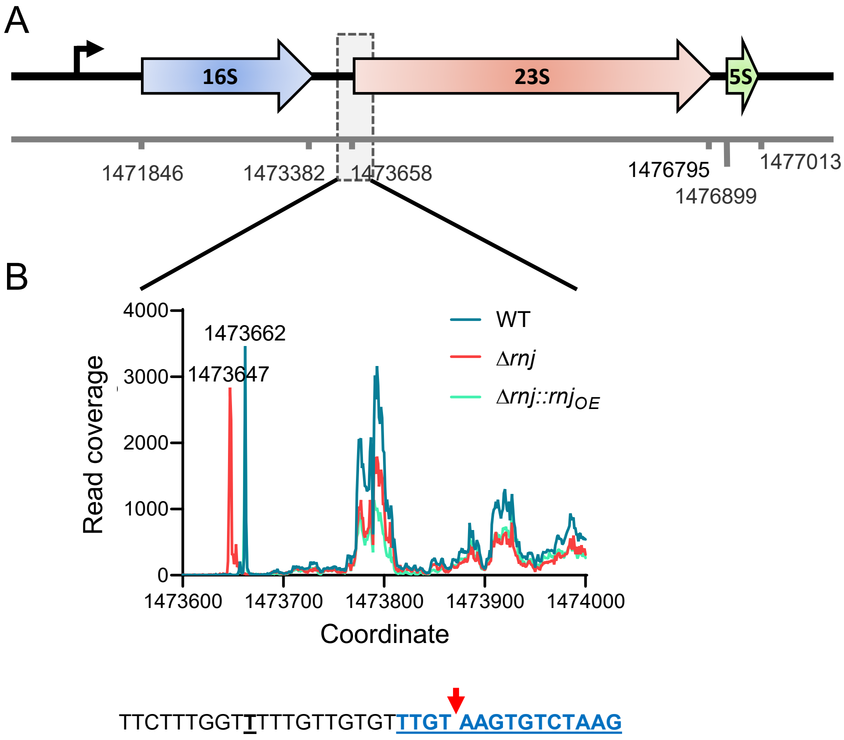

S5 Figure. RNase J contributes to 23S rRNA maturation in Mtb. A. Schematic of the rRNA operon in Mtb. The genome positions in the reference genome NC_000962 are given. The RNase J-dependent processing region of the annotated 23S rRNA is shown in a dashed rectangle. **B.** Read depth of 5' end-mapping libraries is shown for the indicated genome coordinates for Δ*rnj*, WT and Δ*rnj*::*rnj*$_{OE}$ strains. The WT and Δ*rnj* strains contained the empty vector pJEB402. The first part of 23S rRNA sequence is highlighted in underlined bolded blue. We did not detect the annotated 23S 5' end at coordinate 1473658 (the first blue nt). The red arrow shows the identified processed 5' end (genome coordinate 1473662) that predominated in the WT and complemented strains. The underlined black "T" indicates the 5' end (genome coordinate 1473647) that predominated in the Δ*rnj* strain.
