# Supplementary material for: Loss of RNase J leads to multi-drug tolerance and accumulation of highly structured mRNA fragments in Mycobacterium tuberculosis
Source: PLoS Pathog. 2022 Jul 13;18(7):e1010705. doi: 10.1371/journal.ppat.1010705 (PMC9312406; doi:10.1371/journal.ppat.1010705)
Supplement: S6 Fig — (PDF) [file ppat.1010705.s012.pdf]

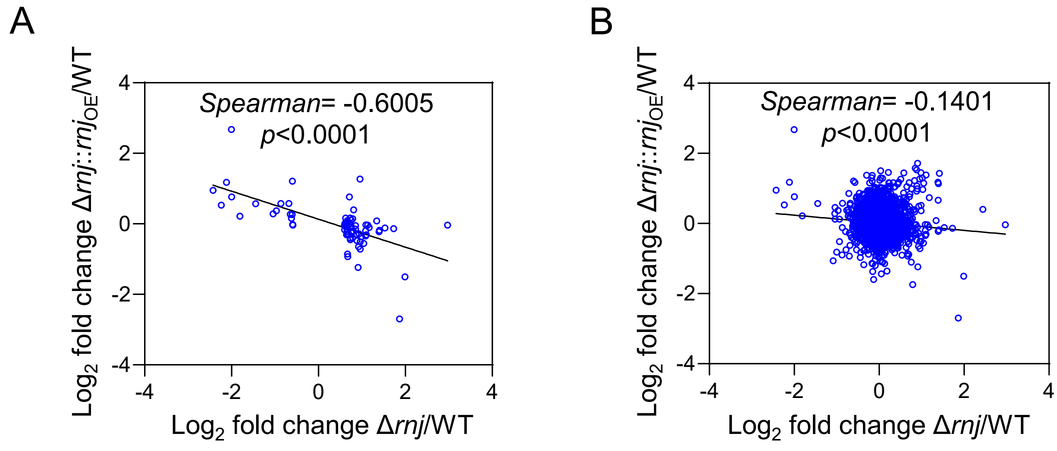

S6 Figure. Genes affected by loss of *rnj* are inversely affected by *rnj* overexpression. DESeq2 analysis was performed independently for Δ*rnj*/WT and Δ*rnj*::*rnj*$_{OE}$/WT and the outputs were compared. The WT and Δ*rnj* strains contained the empty vector pJEB402. In **A**, only those genes that were differentially expressed in Δ*rnj* are shown. In **B** all the genes used in DEseq2 analysis are shown.
