# Supplementary material for: Loss of RNase J leads to multi-drug tolerance and accumulation of highly structured mRNA fragments in Mycobacterium tuberculosis
Source: PLoS Pathog. 2022 Jul 13;18(7):e1010705. doi: 10.1371/journal.ppat.1010705 (PMC9312406; doi:10.1371/journal.ppat.1010705)
Supplement: S7 Fig — (PDF) [file ppat.1010705.s013.pdf]

A

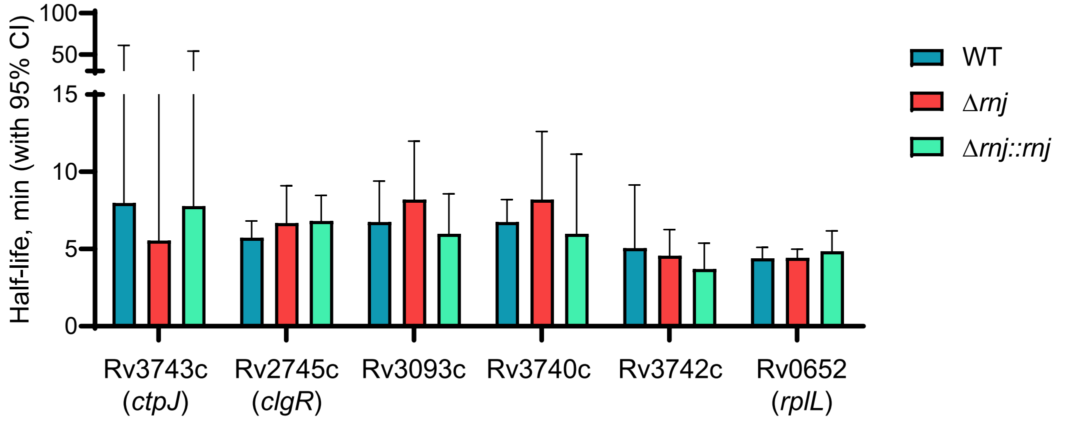

B

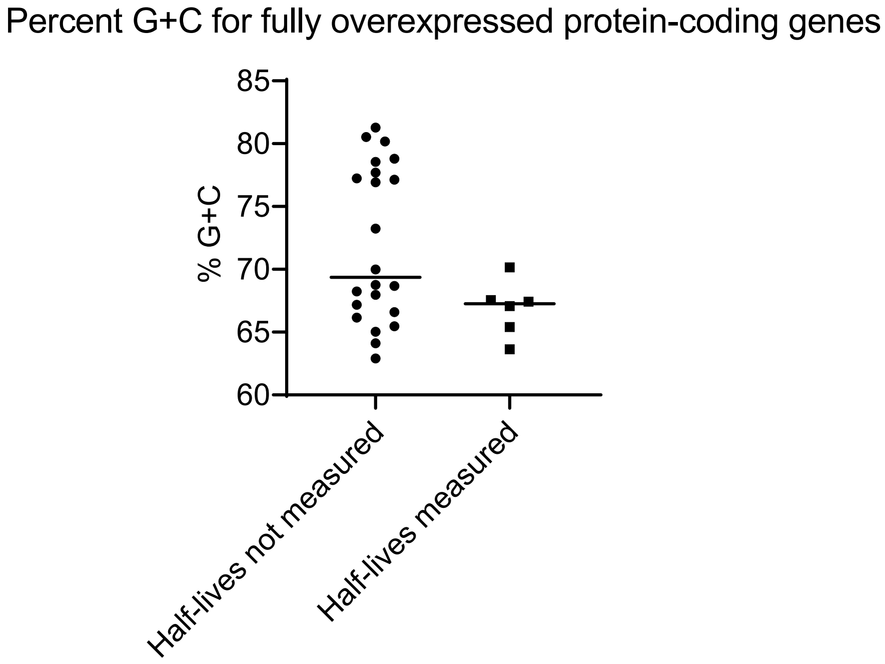

S7 Figure. Some of the genes fully overexpressed in Δ*rnj* do not display increased stability. A. Half-lives of 6 genes fully overexpressed in Δ*rnj* were measured in Mtb mc^{2}6230 WT, Δ*rnj,* and Δ*rnj*::*rnj* strains. **B.** Percent G+C content for the coding sequences of all fully overexpressed genes.
