# Supplementary material for: Loss of RNase J leads to multi-drug tolerance and accumulation of highly structured mRNA fragments in Mycobacterium tuberculosis
Source: PLoS Pathog. 2022 Jul 13;18(7):e1010705. doi: 10.1371/journal.ppat.1010705 (PMC9312406; doi:10.1371/journal.ppat.1010705)
Supplement: S8 Fig — (PDF) [file ppat.1010705.s014.pdf]

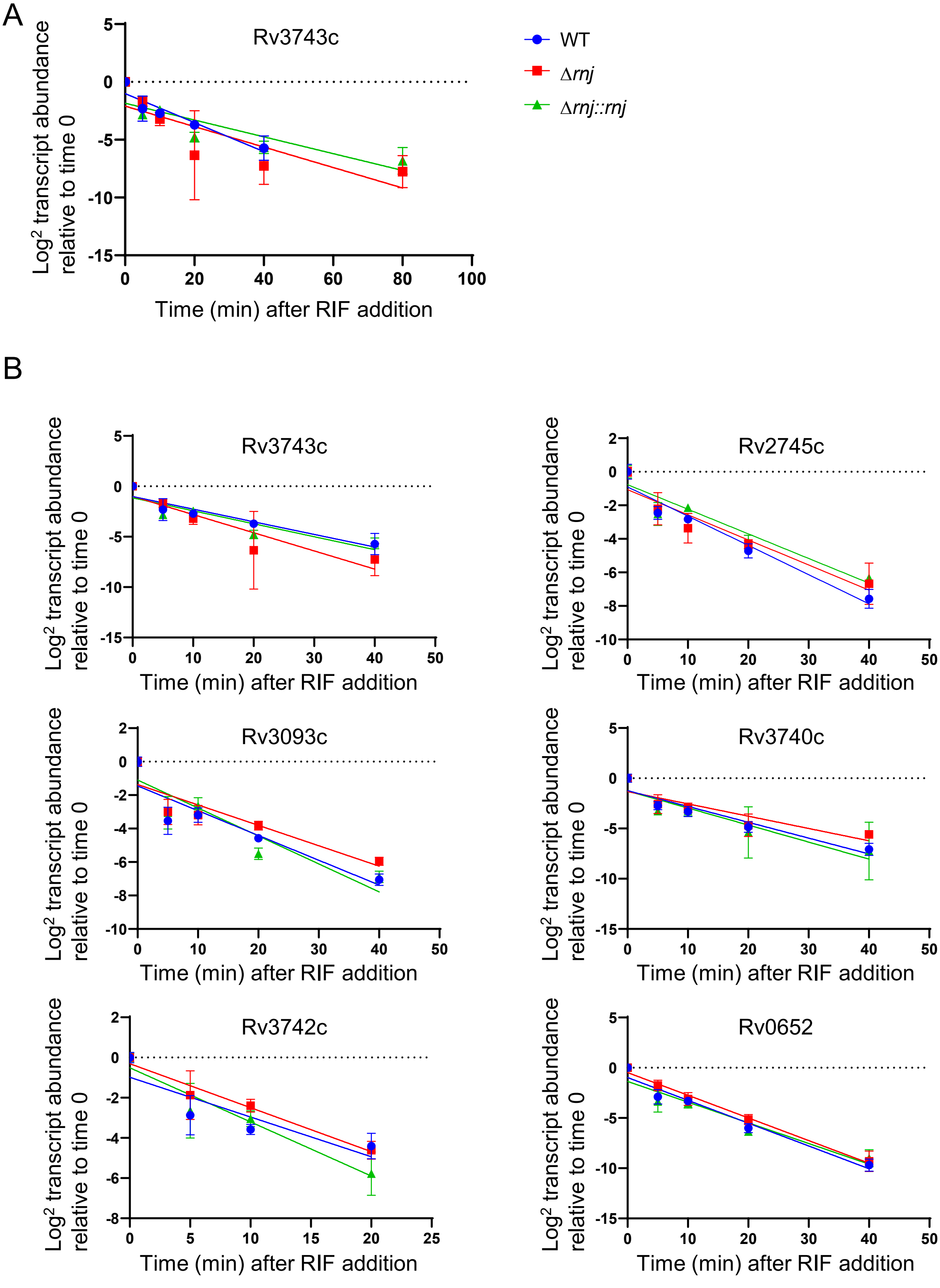

S8 Figure. Decay curves used to calculate half-lives in S7 Fig. Rifampicin (RIF) was added to log-phase Mtb cultures at time 0. Samples were flash-frozen in liquid nitrogen at the indicated time-points. RNA was extracted and transcript abundance measured by quantitative PCR. **A.** An example of a decay curve for which a late time-point (80 min) did not follow the single exponential decay trend seen for the earlier time-points, and for which there were missing data at a late time-point. In such cases the late time-point in question was omitted from half-life calculations. Half-lives of 6 genes fully overexpressed in Δ*rnj* were measured in Mtb mc^{2}6230 WT, Δ*rnj,* and Δ*rnj*::*rnj* strains. **B.** The decay curves used to calculate the half-lives shown in S7 Fig. Note that for Rv3742c, the 40 min time-point was omitted because there were substantial missing data from samples for which the CT values were too high to reliably distinguish from background. The strain key is as in panel A.
