# Supplementary material for: Loss of RNase J leads to multi-drug tolerance and accumulation of highly structured mRNA fragments in Mycobacterium tuberculosis
Source: PLoS Pathog. 2022 Jul 13;18(7):e1010705. doi: 10.1371/journal.ppat.1010705 (PMC9312406; doi:10.1371/journal.ppat.1010705)
Supplement: S9 Fig — (PDF) [file ppat.1010705.s015.pdf]

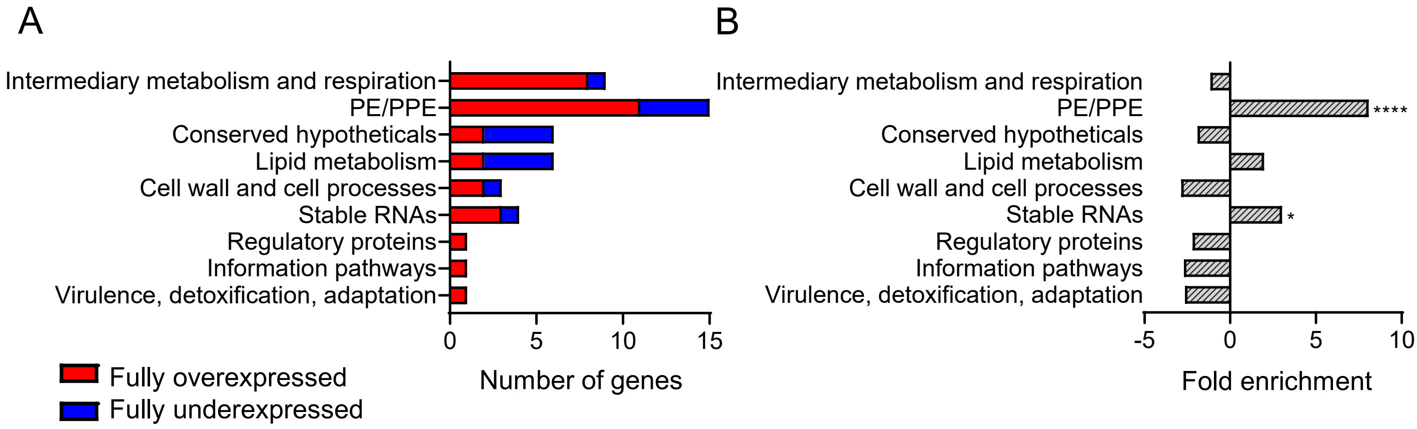

S9 Figure. Genes that are differentially expressed in the absence of *rnj* in the H37Rv background are enriched for stable RNAs and PE/PPE family genes. A. Classification of the fully over- and underexpressed genes by category. **B.** Gene category enrichment for fully over- and underexpressed genes using hypergeometric test. *p<0.05, ****p<0.001.
