# Supplementary material for: Loss of RNase J leads to multi-drug tolerance and accumulation of highly structured mRNA fragments in Mycobacterium tuberculosis
Source: PLoS Pathog. 2022 Jul 13;18(7):e1010705. doi: 10.1371/journal.ppat.1010705 (PMC9312406; doi:10.1371/journal.ppat.1010705)
Supplement: S10 Fig — (PDF) [file ppat.1010705.s016.pdf]

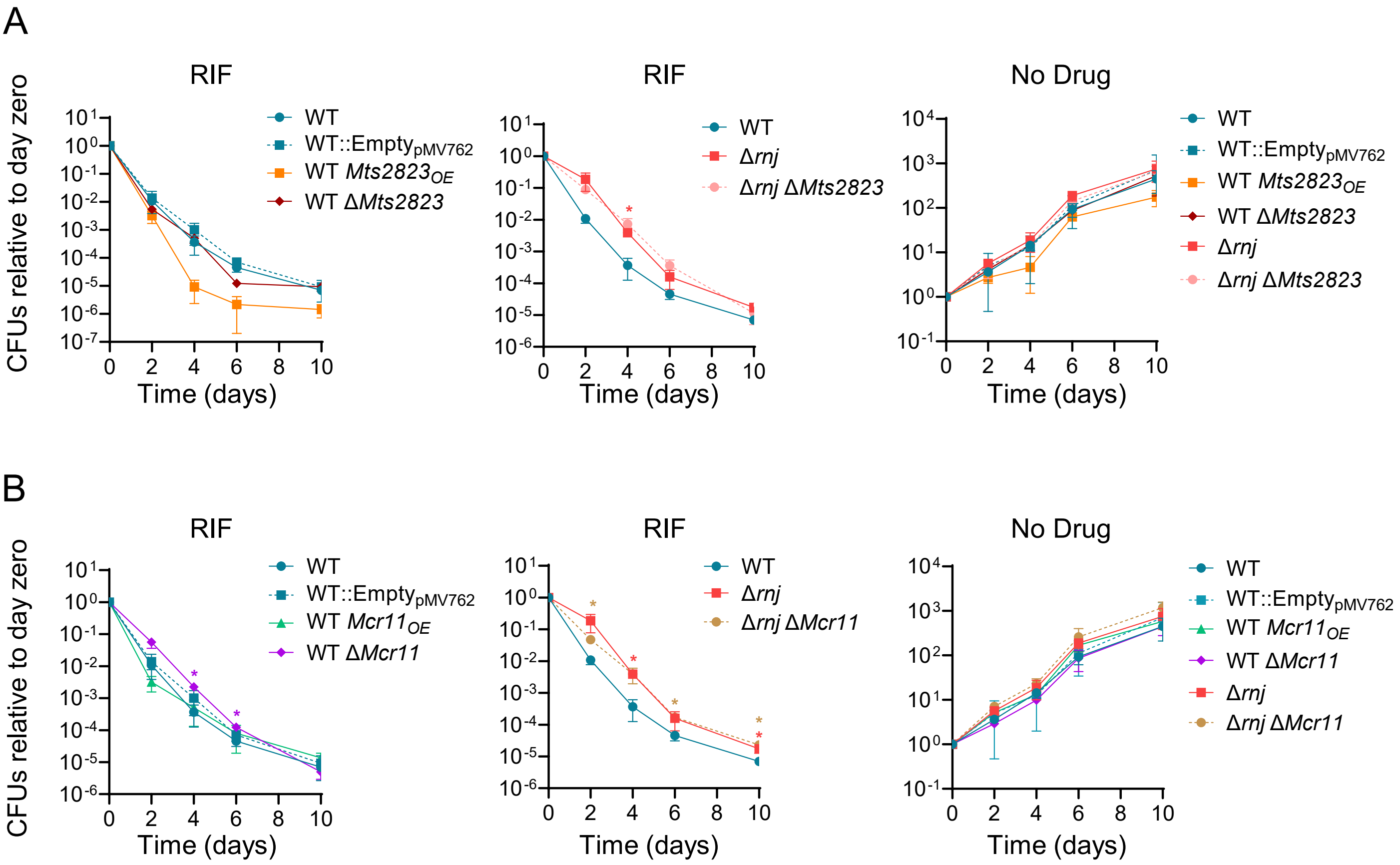

S10 Figure. The sRNAs *Mts2823* and *Mcr11* do not affect RIF tolerance in WT or Δ*rnj* Mtb. Time-killing curves in presence RIF (0.6 μg/mL) for strains with deletion or overexpression of *Mts2823* **(A)** or *Mcr11* **(B)** strains are shown. OE indicates overexpression of the indicated sRNA. Growth in drug-free media was also assessed. *p<0.05, **p<0.001, two-way ANOVA. Red stars: comparison of WT to Δ*rnj*. Violet stars: comparison of WT Δ*Mcr11* to WT. Tan stars: comparison of Δ*rnj* Δ*Mcr11* to WT. The apparent increase in RIF tolerance seen here for the WT Δ*Mcr11* strain was not reproducible.
