# Supplementary material for: Loss of RNase J leads to multi-drug tolerance and accumulation of highly structured mRNA fragments in Mycobacterium tuberculosis
Source: PLoS Pathog. 2022 Jul 13;18(7):e1010705. doi: 10.1371/journal.ppat.1010705 (PMC9312406; doi:10.1371/journal.ppat.1010705)
Supplement: S11 Fig — (PDF) [file ppat.1010705.s017.pdf]

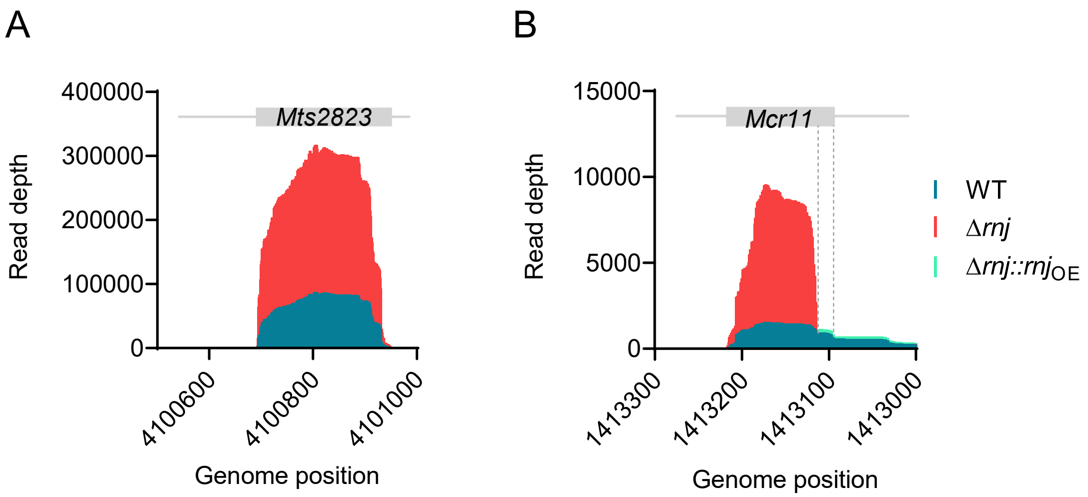

S11 Figure. RNAseq coverage plots of sRNAs *Mts2823* (A) and *Mcr11* (B). Read depth in RNAseq expression libraries is shown. The region of *Mcr11* displaying similar coverage in Δ*rnj* and the WT is denoted with dashed lines. The WT and Δ*rnj* strains contained the empty vector pJEB402.
