# Supplementary material for: Loss of RNase J leads to multi-drug tolerance and accumulation of highly structured mRNA fragments in Mycobacterium tuberculosis
Source: PLoS Pathog. 2022 Jul 13;18(7):e1010705. doi: 10.1371/journal.ppat.1010705 (PMC9312406; doi:10.1371/journal.ppat.1010705)
Supplement: Attachment — Submitted filename: Martini et al response to reviewers.docx [file ppat.1010705.s018.docx]

**Introduction to the revision**

We are pleased to submit a substantially revised version of our manuscript entitled “Loss of RNase J leads to multi-drug tolerance and accumulation of highly structured mRNA fragments in *Mycobacterium tuberculosis*.” Here we describe the presence and nature of mutations in the gene encoding RNase J in clinical Mtb strains, demonstrate that deletion of RNase J leads to increased tolerance to several clinically important drugs, demonstrate that this drug tolerance is dependent upon altered expression of several genes, and show that RNase J has a role in rRNA processing and degradation of structured RNAs. We thank the reviewers for their thoughtful comments and hope you will agree that in addressing those comments we have substantially strengthened the manuscript.

The most substantial criticism of the first submission was that while we showed that downregulation of *ppe50-ppe51* was necessary for the drug tolerance phenotype of an RNase J deletion strain, we did not investigate the impact of *ppe50-ppe51* in a WT background. We therefore made *ppe50-ppe51* deletion and complemented strains in a WT background and found that deletion of *ppe50-ppe51* caused tolerance to RIF and INH. We have therefore shown that reduction or loss of *ppe50-ppe51* expression is both necessary and sufficient for tolerance to these two first-line TB drugs. This finding provides mechanistic insight into the effects of RNase J on drug tolerance, and also has broader implications for the importance of central carbon metabolism in drug tolerance.

We detail the specific changes and respond to each comment in blue below.

**Reviewer's Responses to Questions and author responses**

**Part I - Summary**
Please use this section to discuss strengths/weaknesses of study, novelty/significance, general execution and scholarship.

Reviewer #1: Although there is a growing body of literature on the mechanisms of antibiotic tolerance in model organisms, the specific mechanisms mediating drug tolerance in M. tuberculosis are still poorly understood. In this work, Martini, Hicks and colleagues investigate the role of RNAseJ in multi-drug tolerance in Mtb. Bacterial GWAS studies from the authors and other groups had previously identified (probable loss of function) mutations in RNAseJ as being associated with drug-resistance. Furthermore, Tnseq analyses by Sassetti and colleagues had shown that transposon insertions in RNAseJ are associated with drug tolerance in vitro and in animal infection. Here, the authors verify that lack of RNAseJ, or replacement with a catalytically null mutant is associated with tolerance of Mtb to multiple antibiotics in vitro. They confirm that RNAseJ processes 23S rRNA. Deletion results in up/downregulation of a fairly discrete set of genes including some PPE genes and sRNAs. The authors suggest RNAseJ ‘client mRNAs’ have high GC content and more stable secondary structures. Surprisingly, measuring the half-life of several candidate RNAseJ mRNAs doesn’t show alteration of half-life +/- RNAseJ. Mechanistically, the most interesting experiments are that deletion of two sRNAs, Mts2823 and Mcr11 rescued the RNAse J tolerance phenotype to INH but not RIF, but had no phenotype in WT bacilli. Similarly, since expression of ppe50-ppe51 is decreased in the KO, the showed that OE of the two genes increased drug susceptibility of the deletion mutant to wild-type levels.

Overall, this a carefully performed study that advances mechanistic study of drug tolerance in Mtb and makes a substantial advance for the field. However, a few points of interpretation may require further nuance, and/or lack a critical control.

Reviewer #2: This study verifies the predicted role of Mtb RNaseJ in 23S rRNA maturation and degradation of more stable RNA structures; and provides new evidence for its role in drug susceptibility. Although specific mechanisms are still unknown (e.g., how is SigM activated in the rnj deletion mutant, why are sRNAs specific for INH, how exactly the loss of RNase J increases drug tolerance), this well-presented and rigorously conducted work provides good foundation for future studies.

Reviewer #3: This manuscript investigates the role of RnaseJ in Mtb in drug tolerance by characterizing an RnaseJ deletion strain of H37Rv and an auxotrophic strain of Mtb. The motivation for the study is the prior finding that WGS of clinical Mtb strains has revealed mutations in the gene encoding RnaseJ in drug resistant strains. The RnaseJ ko has the following phenotypes:

1) Loss of RnaseJ attenuates the rate of killing of Mtb by INH, Rif, Oflox, and ethambutol and these phenotypes are complemented by the wild type gene. The magnitude of the effect is most dramatic with INH at early time points.
2) Small colony size of the RnaseJ KO
3) RNA sequencing of RnaseJ ko revealed transcripts that are altered, and mapping of the RNAseq reads reveals that specific regions of transcripts are overabundant, rather than entire mRNAs, and these regions have different predicted secondary structure.
4) The authors go on to interrogate several differentially abundant transcripts and sRNAs and show suppression of the antibiotic sensitivity phenotype for some, but overexpression of these targets was not sufficient to produce drug sensitivity.

Major Comments:

Experimental

Overall, the study is rigorously performed, and I have very few experimental criticisms except for some minor technical points given below. Appropriate controls and statistical tests are applied, and the scientific quality is very high. The major experimental challenge is the magnitude of some of the phenotypes observed, which in many cases are subtle. The most significant antibiotic sensitization is to INH, but in all cases the effects are mild but statistically significant.

We agree with the reviewer that most of the effects we see are small. However, there is a growing body of literature reporting that subtle differences in drug sensitivity can have clinically significant consequences. For example, small differences in MIC were shown to affect TB cure rates (N Engl J Med 2018; 379:823-833. DOI: 10.1056/NEJMoa1715849), and strong positive selection has been reported for mutations causing drug tolerance of similar magnitude to what we report here (Nat microbiol 2018; 3,9: 1032-1042. doi:10.1038/s41564-018-0218-3 and Nat Genet 2013; 45: 1183–1189. doi:10.1038/ng.2747). We therefore believe that further investigation of potential stepping-stone mutations is important to improve our understanding of how drug resistance develops in the clinic.

The effects of RnaseJ loss of RNA stability are convincing, but in most cases shown are due to persistence of a certain segment of the RNA within the transcript**. Although this is plausibly attributed to the structure of that region, I think this analysis could be more extensive. The overabundant regions are quite short in most cases. Are there secondary structures of the RNAs that can be predicted in those segments? Have unannotated small RNAs been excluded as the cause of these overabundant regions as several other annotated sRNAs are found?**

The overabundant regions that we defined in the RNase J deletion strain are in annotated coding sequences and therefore do not overlap with annotated sRNAs, as sRNA identification pipelines usually exclude coding sequences. We cannot exclude the possibility that some of these regions are in fact functional sRNAs. Testing this possibility is technically challenging because one must ensure that manipulations of the putative sRNA do not affect expression of the overlapping coding sequence. It would certainly be interesting to investigate this idea in future work. However, in the absence of any evidence of function at this point, the simplest explanation is that the overabundant regions are degradation products.

We have performed additional analyses of overabundant regions as suggested, which we show in the revised Figure 4 (previously Figure 3) and describe in the text.

Given that most of the RnaseJ mutations found in clinical strains are not predicted to be null, it would be useful to engage in some structural modeling of the mutations to attempt to predict their effect.

We have performed analyses to predict the effects of the clinical mutations, and the results are shown in Figure 1, Table 1, and Table S3.

Interpretation of findings

The major challenge of distilling the findings into a model of how RnaseJ affects antimicrobial sensitivity is the pleiotropic nature of the effects of RnaseJ deletion. This is not really a criticism, but a reflection of attributing specific mechanisms to pathways that are global. As such, the findings do not yield a clear picture of what downstream pathways are controlling this antibiotic phenotype. It is possibly a reflection of a globally altered cell, including possible changes in permeability due to cell wall alteration, metabolic changes, etc. I am sympathetic to the challenge, but the firmest conclusion is that loss of RnaseJ globally affects antibiotic sensitivity, without a clear mechanism of how this occurs. This lack of downstream mechanism somewhat limits the broad impact of the findings.

We expect that our study will be of broad interest because we demonstrate for the first time that RNA degradation pathways affect drug sensitivity, and we show that altered expression of specific genes is required and in some cases sufficient to produce drug tolerance.

The reviewer highlights a good point regarding the challenges of understanding pleiotropic effects. Indeed, recent studies of various bacteria have revealed mutations with pleiotropic effects that alter antibiotic susceptibility ([10.1128/mSystems.00396-20](https://doi.org/10.1128/msystems.00396-20); [10.1128/mBio.00663-19](https://doi.org/10.1128/mbio.00663-19); [10.1038/nature13469](https://doi.org/10.1038/nature13469); [10.1128/AAC.02103-19](https://doi.org/10.1128/aac.02103-19); [10.1128/AAC.01431-21](https://doi.org/10.1128/aac.01431-21); [10.1099/mgen.0.000623](https://doi.org/10.1099/mgen.0.000623); [10.1038/s41467-022-30272-0](https://doi.org/10.1038/s41467-022-30272-0)). For example, Bellerose and colleagues ([10.1128/mBio.00663-19](https://doi.org/10.1128/mbio.00663-19)) showed that *M. tuberculosis* mutants unable to use glycerol as a carbon source had increased tolerance to several antibiotics, and the importance of this phenotype as a pathway to high-level drug resistance was highlighted by the prevalence of such mutants among drug-resistant clinical strains. Furthermore, the effect of these mutations was greatest when considering susceptibility to pyrazinamide, a drug for which the mechanism of action remains unclear, likely because the drug has pleotropic effects on mycobacterial physiology. Other genes shown to affect drug sensitivity via perturbations to central carbon metabolism (which inherently causes pleotropic effects) in mycobacteria include succinate dehydrogenase ([10.1128/mSystems.00396-20](https://doi.org/10.1128/msystems.00396-20)) and *ppe50-51* ([10.1128/mBio.00663-19](https://doi.org/10.1128/mbio.00663-19)).

These findings highlight a challenge for the field: how can we fully elucidate the mechanisms of drug sensitivity effects in mutants that have pleiotropic consequences? Doing so will not be trivial, particularly for situations like RNase J disruption where multiple classes of drugs are affected and where multiple mechanisms appear to be involved in both drug-specific and non-specific ways. We plan to address this in future studies, and expect that publishing our current study will lay a foundation for other groups to tackle these challenging questions as well.

**Part II – Major Issues: Key Experiments Required for Acceptance**
Please use this section to detail the key new experiments or modifications of existing experiments that should be absolutely required to validate study conclusions.

Generally, there should be no more than 3 such required experiments or major modifications for a "Major Revision" recommendation. If more than 3 experiments are necessary to validate the study conclusions, then you are encouraged to recommend "Reject".

Reviewer #1: 1. I’m not sure I agree with the interpretation with regards to whether the deletion mutant phenotype isn’t via lag phase modulation or doesn’t influence persister levels. As per the classic Balaban paper (PMID: 25043002), which incidentally, hasn’t been cited, lag time is strongly associated with persistence. Furthermore, the growth curve in Fig. 2A, does seem to show a difference in growth rates (although subtle, it is a log10 scale). The experiments meant to exclude lag phase aren’t totally convincing: The expt in 2D goes some way to address the issue, but even at day 4, the OD was still only ~0.01 and not really log phase. Ideally, repeating a kill curve when cultures are > OD 0.1 would help resolve the issue.

The reviewer is correct that we cannot fully rule out the possibility that a lag phase defect or increased persister frequency contribute to the observed drug tolerance in the RNase J deletion strain. We concluded that persister levels were similar in the WT and RNase J deletion strains based on the persister plateau shown in Figure 2B. It is possible that if we used a different drug combination or different assay, we would see differences in persister levels. We have modified the language on lines 315-319 to reflect these possibilities and added a reference to the Balaban paper.

The RNase J deletion strain indeed has a longer lag phase than the WT and complemented strains. We did not detect differences in growth rate once the strains reached log phase. Importantly, we also observed drug tolerance in the RNase J deletion strain in minimal media where there was no detectable lag phase defect (Fig. S4). We then performed the experiment shown in what is now Figure 3B-C to determine if the lag phase differences in 7H9 contributed to tolerance in that condition. It appears this figure was not fully clear. At day 0, the cultures were diluted to an OD of 0.02 and were therefore not in log phase. They were then grown for 4 days. Each day, an aliquot was removed and treated with INH. By day 4, the cell density prior to INH addition was approximately 2 x 10^9^ cfu/ml. The experiment therefore encompassed various growth stages including log. We revised the figure legend to better explain this experiment.

2. A puzzling result for me is with regards to the upregulation of certain transcripts in the deletion mutant. The authors nicely show that RNAseJ targets high GC-rich transcripts, which nicely fits the upregulated transcripts. Rather surprisingly, however, the half-life of some of these transcripts is not altered in the deletion mutant. That doesn’t make sense to this reader. Are the data for Fig. 3E/F for all upregulated transcripts, or for just the partially upregulated (which appear to be degraded by RNAseJ)? One would expect the partially upregulated transcripts to have a different GC content to the fully upregulated transcripts (i.e. those whose half-life is not altered).

The simplest model that fits our observations is that in the absence of RNase J some transcripts and transcript fragments accumulate to higher steady-state abundances because they are degraded more slowly, while some transcripts change in abundance due to altered transcription rates that occur as downstream consequences of the loss of RNase J. We measured the half-lives of several fully-overexpressed genes expecting that those that were RNase J targets would have longer half-lives, while those that were upregulated transcriptionally would have unchanged half-lives. None of the six fully-overexpressed transcripts we tested had longer half-lives in the RNase J deletion mutant (Fig. S7), suggesting that they are overexpressed due to increased transcription rather than slower degradation. Notably, there is a bias with respect to the transcripts that we tested. We tested qPCR primer sets for a longer list of upregulated genes, and only measured half-lives for a subset of genes for which we found primer pairs with high efficiency (90-110%). This excluded the 9 PE-PGRS genes that were upregulated in the absence of RNase J. PE_PGRS genes are GC-rich and contain repetitive regions and as a result it is more difficult to find high-efficiency qPCR primer pairs to measure them. However, our other data suggest that GC-rich transcripts are exactly those that are most likely to be degraded by RNase J. We have summarized the GC contents of genes for which we did and did not measure half-lives in a new figure panel, S7B. Meanwhile, the fully upregulated genes include at least two predicted to affect transcription (*clgR* and MTS2823) and one ribosomal protein gene, and we see evidence of SigM activation. It therefore seems likely that many of the genes fully upregulated in the absence of RNase J are upregulated by increased transcription as an indirect consequence of RNase J deletion, rather than by decreased degradation. Notably, *clgR* is a regulator of proteolysis by the Clp system, which means there may be functionally important changes in protein levels that are not reflected in the transcriptome. We have edited the text to clarify these points (lines 385-392).

The data in the previous Fig 3 E-F (now figure 4 F-G) are from 5’-end-directed RNAseq libraries and reflect a mixture of full-length transcripts and degradation products. We have added a new figure panel, Fig. 4E, showing analysis of the predicted secondary structures of overexpressed vs. non-overexpressed regions identified in the standard RNAseq dataset that was analyzed in panels A-D.

3. The ppe50-ppe51 experiment lacks a control. Much like the former experiment with the sRNAs, the drug tolerance should also be performed in OE on WT background. Otherwise the statements on lines 361/399 that the phenotype in part explains drug tolerance in absence of RNAseJ cannot be made. Indeed, the Bellerose data suggest that this phenotype doesn’t require RNAseJ deletion.

We have added the appropriate control, which is deletion of *ppe50-51* in a WT background (Figure 6B). We found that loss of *ppe50-51* leads to RIF and INH tolerance. This is consistent with the idea that the reduced expression of *ppe50-51* in the RNase J deletion mutant is part of the mechanism of tolerance in that strain.

Reviewer #2: None

Reviewer #3: I think the aspects of the paper that could be improved are the analysis of the possible structures of the RNA segments and more information about the predicted effect of RNAseJ mutations in clinical strains, but these experiments are secondary to the editorial judgement about whether the findings are of broad interest given the limitations noted above.

We have added panels to Figure 1 showing where the clinical point mutations map on a crystal structure, and a table (Table 1) summarizing the numbers and types of mutations and their predicted effects when data was available from studies in other bacteria. We added more detailed information about the predicted effects of individual mutations to Table S3. We have also added a sequence alignment (Figure S2) to show which mutations occurred at conserved positions.

We have performed additional analyses of RNA sequences overexpressed in the RNase J mutation, which we show in the revised Figure 4 (previously Figure 3) and describe in the text.

**Part III – Minor Issues: Editorial and Data Presentation Modifications**
Please use this section for editorial suggestions as well as relatively minor modifications of existing data that would enhance clarity.

Reviewer #1: 1. The lack of full processing of 23S rRNA was intriguing. I’m wondering whether at least some of the phenotype is via effects on translational speed. Addressing this experimentally is beyond the scope of the current work, but may be worth discussing in the Discussion?

We agree this is an interesting question. The location of the 5’ end of the 23S ribosome in the 50S subunit suggests it may play a role in ribosome assembly, and this idea warrants further study. We have added a paragraph to the discussion regard this topic.

2. In the text, Fig. 4 is called out as Fig. 6

We have corrected this error.

3. The reference formatting is off, including, but not limited to unusually abbreviated journal names. This should be corrected.

We have corrected this error.

Reviewer #2: In the RNA half-life measurements, 160 min time point was excluded because “it did not follow the initial exponential decay trend”. How many times was this experiment repeated and was this observation consistent? Is there any mechanistic explanation for this observation? It would be good to include graphs in SI that show these data.

In *M. smegmatis* (which was not used in this work) we consistently observe that following addition of RIF there is a period of exponential decay, followed by a period in which decay is much slower or no decay is evident. We occasionally observe this in Mtb. This has been reported in *E. coli* by some groups (<https://doi.org/10.7554/eLife.13065.001> and <https://doi.org/10.15252/msb.20145794>) but not others (<https://doi.org/10.1093/nar/gkt1150> for example). We do not have a mechanistic explanation for this phenomenon, but we have ongoing work in the lab to investigate it. The half-life experiments were performed twice for the genes shown in Fig 4D and once for the genes shown in Fig S7.

We found an error in the text regarding the half-life timepoints used and have corrected it and added a better explanation of the circumstances in which we omit timepoints from half-life calculations. In addition to removal of late timepoints that didn’t follow the exponential decay trend, we also removed some late timepoints because there were missing CT values. We added the decay curves to the supplemental material as suggested, including a panel demonstrating the rationale for omission of timepoints (Fig S7).

2021 WHO TB report should be cited, not the one from 2020, since the report from 2020 shows data from year 2019, not 2020, as the authors claim in the intro. Also, TB is not top 10 cause of death anymore. The report from 2021 states that it was 13th leading cause of death in 2019 (Fig. 7), while report from 2020 shows data for year 2016, when TB was 10th (Fig. 4.14). The text should be updated to reflect newer data and more recent WHO report. Note that WHO report from 2021 was mentioned in the first paragraph, but it is not listed as a reference.

We appreciate the reviewer identifying these errors, and we have corrected them.

Line 63 – Period is missing before “For example…”
Line 106 – liter should be L
Line 107 – “Tween 80” instead of “tween”
Line 342 – Fig 6 should be Fig 4

We appreciate the reviewer’s sharp eye!

Reviewer #3: Figure 1A. given the compelling finding that RnaseJ mutations are found in clinical drug resistant strains, I think this panel could be better presented. The mutation types are difficult to see.

We have added panels showing the mutations mapped onto a crystal structure (Figure 1), as well as a table summarizing the mutation types, numbers, and predicted effects when supporting data were available.

Figure 2: the colony size phenotype: is this conserved in the non-auxotrophic strain background and is it complemented by the wild type rnaseJ?

Yes, and yes. Unfortunately, we did not take pictures of plates of the complemented strain in the auxotroph background or of any of the H37Rv strains. We therefore removed the colony size data from Figure 3.

For the complementation experiments using the H86A mutation, it should be noted that the lack of complementation, although consistent with a necessity for RnaseJ activity, could also be due to protein instability unless an antibody is available to confirm expression.

We have added this qualification to lines 284-287.

Many of the references are misformatted and missing journal names and other information.

We have corrected this error.

The delta symbol in front of italicized bacterial gene names should not be also italicized

We have corrected this error.
